# Investigating repetitive transcranial magnetic stimulation on cannabis use and cognition in people with schizophrenia

**DOI:** 10.1038/s41537-022-00210-6

**Published:** 2022-02-24

**Authors:** Karolina Kozak Bidzinski, Darby J. E. Lowe, Marcos Sanches, Maryam Sorkhou, Isabelle Boileau, Michael Kiang, Daniel M. Blumberger, Gary Remington, Clement Ma, David J. Castle, Rachel A. Rabin, Tony P. George

**Affiliations:** 1 Institute of Medical Science (IMS), University of Toronto, Toronto, ON Canada; 2 Addictions Division, Centre for Addiction and Mental Health (CAMH), Toronto, ON Canada; 3 Krembil Centre for Neuroinformatics, CAMH, Toronto, ON Canada; 4 Centre for Complex Interventions, CAMH, Toronto, ON Canada; 5 Addiction Imaging Research Group, Campbell Family Mental Health Research Institute, CAMH, Toronto, ON Canada; 6 Division of Brain and Clinical Translation, Department of Psychiatry, University of Toronto, Toronto, ON Canada; 7 Schizophrenia Division, CAMH, Toronto, ON Canada; 8 Temerty Centre for Therapeutic Brain Intervention, CAMH, Toronto, ON Canada; 9 Campbell Family Mental Health Research Institute, CAMH, Toronto, ON Canada; 10 Division of Biostatistics, Dalla Lana School of Public Health, University of Toronto, Toronto, ON Canada; 11 Douglas Mental Health Research Centre, and Department of Psychiatry, McGill University, Montreal, QC Canada

**Keywords:** Schizophrenia, Working memory

## Abstract

Cannabis use disorder (CUD) occurs at high rates in schizophrenia, which negatively impacts its clinical prognosis. These patients have greater difficulty quitting cannabis which may reflect putative deficits in the dorsolateral prefrontal cortex (DLPFC), a potential target for treatment development. We examined the effects of active versus sham high-frequency (20-Hz) repetitive transcranial magnetic stimulation (rTMS) on cannabis use in outpatients with schizophrenia and CUD. Secondary outcomes included cannabis craving/withdrawal, psychiatric symptoms, cognition and tobacco use. Twenty-four outpatients with schizophrenia and CUD were enrolled in a preliminary double-blind, sham-controlled randomized trial. Nineteen participants were randomized to receive active (*n* = 9) or sham (*n* = 10) rTMS (20-Hz) applied bilaterally to the DLPFC 5x/week for 4 weeks. Cannabis use was monitored twice weekly. A cognitive battery was administered pre- and post-treatment. rTMS was safe and well-tolerated with high treatment retention (~90%). Contrast estimates suggested greater reduction in self-reported cannabis use (measured in grams/day) in the active versus sham group (Estimate = 0.33, *p* = 0.21; Cohen’s *d* = 0.72), suggesting a clinically relevant effect of rTMS. A trend toward greater reduction in craving (Estimate = 3.92, *p* = 0.06), and significant reductions in PANSS positive (Estimate = 2.42, *p* = 0.02) and total (Estimate = 5.03, *p* = 0.02) symptom scores were found in the active versus sham group. Active rTMS also improved attention (Estimate = 6.58, *p* < 0.05), and suppressed increased tobacco use that was associated with cannabis reductions (Treatment x Time: *p* = 0.01). Our preliminary findings suggest that rTMS to the DLPFC is safe and potentially efficacious for treating CUD in schizophrenia.

## Introduction

Globally, 21 million people suffer from schizophrenia^1^. The high prevalence of cannabis use in schizophrenia is well-established, with lifetime rates of 53.7%^2^. A meta-analysis found that 25% of people with schizophrenia have cannabis use disorder (CUD)^3^ compared to ~3% in general population^4^. CUD is more common among first-episode male versus female patients (40.8% vs. 16.9%, respectively)^5^.

Strong evidence suggests that cannabis use exacerbates psychiatric symptoms, and is a risk factor for schizophrenia^6,7^. The active ingredient in Cannabis (e.g., delta-9-tetrahydrocannabinol, THC) increases positive symptoms^8^, which relates to increased striatal dopaminergic activity^9^. It has been suggested that chronic cannabis use may produce a long-term psychosis (known as Substance-Related Exogenous Psychosis; SREP^10^), but this is known to be a psychosis that presents in atypical manner to schizophrenia with multimodal sensory hallucinations and is likely present in those individuals with a genetic predisposition to cannabis-related psychosis^11–13^. Interestingly, data have been mixed on the effects of cannabis on negative symptoms. Patients report that cannabis use reduces anxiety and depression^14,15^. In contrast, THC worsens negative symptoms, due to its impact on frontal and striatal dopaminergic networks^16^. Moreover, the effects of cannabis use on cognitive function in schizophrenia appear mixed^17^. However, intravenous THC impairs verbal learning and memory^18^, while extended cannabis abstinence improves this outcome selectively in schizophrenia^19^. This is important since cognition predicts functional outcomes^20^.

Managing CUD in schizophrenia remains clinically challenging, with no approved treatments^21^. Nonetheless, promising pharmacotherapies, behavioral, and neuromodulatory therapies are being investigated. For instance, retrospective studies^22,23^ and two randomized controlled trials (RCTs)^24,25^ using atypical antipsychotics, particularly clozapine, found beneficial effects on psychosis and cannabis use. RCTs evaluating behavioral therapies (e.g., contingency management (CM), cognitive behavioral therapy) for schizophrenia and CUD, are limited as typically patients with co-occurring mental illness are excluded in CUD trials, and most studies evaluated the efficacy on cannabis use rather than CUD, or early psychosis versus schizophrenia^26,27^. Notably, our group demonstrated that a 28-day cannabis abstinence paradigm with CM produced high rates of abstinence (~43%) in cannabis-dependent schizophrenia outpatients, comparable to cannabis-dependent non-psychiatric controls (~55%)^28^.

Neuromodulation is a potential treatment option for CUD, including repetitive transcranial magnetic stimulation (rTMS);^29,30^ this entails an electromagnetic coil placed against the scalp to produce magnetic field pulses in rapid succession to targeted brain regions. Most studies stimulate the dorsolateral prefrontal cortex (DLPFC) to modulate cortical excitability and strengthen cognitive control^31,32^. High-frequency rTMS has shown promise in reducing drug craving^33,34^ and use for several substance use disorders (SUDs) including CUD^29–31,34–38^. Several factors differ between studies including the number of rTMS sessions, site of stimulation, presence of a sham control, whether left, right or bilaterally administered, sample size, and presence of comorbid psychiatric illnesses. However, a consensus exists that targeting the DLPFC is most effective, given its role in inhibitory control of reward circuits, including mesocorticolimbic dopamine systems^39,40^. Moreover, given that this area is important in executive function and commonly impaired among individuals with SUDs resulting in relapse, most studies apply high-frequency rTMS (e.g., 10–20 Hz) to the DLPFC to decrease craving. One study examined the efficacy of rTMS for CUD using a single 10-Hz rTMS session to the DLPFC to non-treatment-seeking individuals with CUD^41^. No significant changes in craving were found between active and sham groups. Recently, this group completed an open-label safety trial applying 20-sessions (10-Hz) to DLPFC over 2 weeks to nine individuals with CUD; only three completed the trial^42^. Thus, further research is warranted. Given that patients experience positive, negative and cognitive deficits, finding an optimal rTMS protocol that targets all three symptoms is important^43^. We adapted our previous rTMS treatment paradigms for tobacco use in schizophrenia^44,45^ towards treating CUD, clinical symptoms, and cognitive deficits in schizophrenia.

Our primary objective was to determine the effects of 4 weeks of active (20-Hz) versus sham rTMS treatment applied bilaterally to the DLPFC on cannabis use in schizophrenia. Secondary objectives examined rTMS on cannabis craving/withdrawal, psychotic symptoms, and cognition. We hypothesized that rTMS would significantly reduce cannabis use, craving/withdrawal, positive and negative symptoms, and improve cognition.

## Results

### Participants

**Fig. 1:**
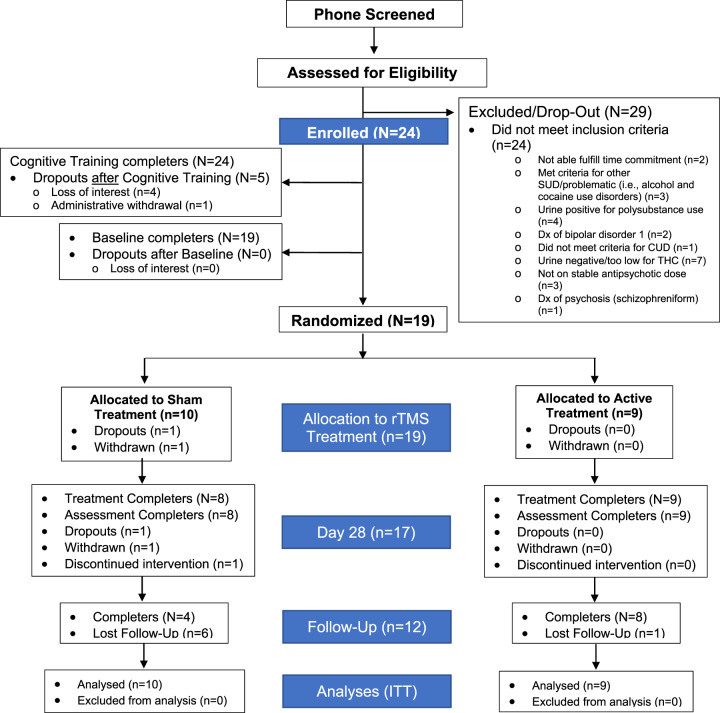
CONSORT Diagram. CONSORT diagram for all participants involved in the study from the first point of contact via phone-screen to randomization, follow-up, and analysis. Abbreviations: CONSORT, consolidated standards of reporting trials; dx, diagnosis; ITT, intention-to-treat analysis; SUD, substance use disorder.

Ninety-nine individuals were telephone screened; fifty-three completed in-person assessments. Twenty-nine individuals did not meet inclusion criteria (see CONSORT Diagram, Fig. 1). Twenty-four patients were enrolled, and completed cognitive training. Reasons for dropouts were loss of interest (*n* = 4) and administrative withdrawal (*n* = 1). This resulted in 19 participants completing baseline, all of which were randomized into active (*n* = 9) or sham (*n* = 10) treatment. All randomized participants were included in the intention to treat (ITT) analysis^46^.

There were no drop-outs in the active group during treatment, with all 9 participants completing treatments and assessments. Eight of these participants completed Day 56. In the sham group, one participant was withdrawn (due to use of study payments for heavy drinking) during Week 3; another sham group participant completed 6 treatments, and then requested to stop receiving treatment and continued with study assessments per ITT design. Thus, in the sham group, *n* = 8 participants all treatment sessions. Four participants completed Day 56, while *n* = 6 did not: dropout (*n* = 1), administrative withdrawal due to protocol violations (*n* = 2), lost to contact (*n* = 1), and scheduling problems (*n* = 2).

Demographic features are listed in Table 1. We compared treatment groups to determine whether demographic characteristics should be controlled for in the analyses. Demographic, clinical and substance use characteristics were comparable between treatment groups. Baseline cognitive performance on the majority of tasks were comparable between treatment groups.

**Table 1:** Demographic, baseline clinical and substance using characteristics.

	Active (*n* = 9)	Sham (*n* = 10)
Age (years)	34.78 ± 3.45	29.10 ± 1.70
Race (C/A/H/O^b^/M)^a^	5/3/1/0/0	4/3/0/1/2
Gender (M/F)	9/0	9/1
Education (years)	13.56 ± 0.87	12.35 ± 0.52
WTAR IQ Score	105.89 ± 3.08	105.20 ± 1.88
TOMM (Trial 2)	49.44 ± 0.24	49.90 ± 0.10
Diagnosis (schizophrenia/schizoaffective disorder)	7/2	7/3
Age of Diagnosis	23.89 ± 1.57	20.40 ± 2.07
Antipsychotics(ATYP/TYP/Both)^a^	6/1/2	7/3/0
PANSS (Positive)	13.56 ± 3.32	10.60 ± 3.53
PANSS (Negative)	15.44 ± 4.67	14.10 ± 4.61
PANSS (General)	25.11 ± 4.43	23.90 ± 4.20
PANSS Total	54.11 ± 9.69	48.60 ± 4.79
C-SSRS	0.67 ± 0.29	0.40 ± 0.31
CDSS	2.33 ± 0.83	1.90 ± 0.64
Tobacco smoker (Yes/No)	8/1	8/2
Expired carbon monoxide level (ppm)	9.33 ± 2.48	15.60 ± 4.80
Cigarettes per day	7.16 ± 2.72	8.72 ± 2.95
FTND	2.56 ± 0.65	3.90 ± 1.04
Alcoholic drinks per day	0.46 ± 0.18	0.26 ± 0.18
AUDIT^c^	3.60 ± 1.08	4.00 ± 0.97
Caffeinated drinks per day	2.44 ± 0.59	2.36 ± 0.76
% Tobacco mixed with cannabis	23.89 ± 7.06	18.20 ± 6.16
Age of first cannabis use	16.33 ± 0.93	15.90 ± 0.72
Age of first regular cannabis use	19.33 ± 0.99	17.20 ± 0.84
# of Cannabis quit attempts	1.67 ± 0.47	2.40 ± 1.23
Method of administration (joint/pipe/blunt/bong/more than one)	5/0/1/0/3	3/1/0/2/4
CUD Severity (moderate/severe)	4/5	4/6
MCL	8.00 ± 0.24	7.70 ± 0.15
CUDIT-R	16.11 ± 1.91	18.10 ± 2.04
URICA	8.30 ± 0.85	8.81 ± 0.79
Joint years	10.37 ± 2.61	6.59 ± 1.47
Cannabis grams per day	0.77 ± 0.13	0.85 ± 0.19
NarcoCheck (ng/mL)	418.67 ± 76.86	391.80 ± 73.81
MWC	10.56 ± 2.44	6.90 ± 0.99
MCQ1	8.33 ± 1.79	8.70 ± 1.89
MCQ2	9.56 ± 1.93	10.80 ± 1.87
MCQ3	12.11 ± 2.00	11.60 ± 2.01
MCQ4	11.78 ± 2.18	13.50 ± 1.79
MCQ Total	41.78 ± 6.78	44.60 ± 7.13
ASI-D	0.11 ± 0.02	0.16 ± 0.03

Values given in means ± standard error.

*A* African American, *ASI-D* Addiction Severity Index-Composite Score for Drug Use, *ATYP* atypical, *AUDIT* Alcohol Use Disorders Identification Test, *C* Caucasian (non-Hispanic), *CDSS* Calgary Depression Scale for Schizophrenia, *C-SSRS* Columbia Suicide Severity Rating-Scale, *CUD* cannabis use disorder, *CUDIT-R* Cannabis Use Disorder Identification Test-Revised, *FTND* Fagerstrom Test for Nicotine Dependence, *H* Hispanic/Latino, *M* More than one race, *MCL* Marijuana Contemplation Ladder, *MCQ* Marijuana Craving Questionnaire, *MWC* Marijuana Withdrawal Checklist, *ng/mL* nanograms per milliliter, *O* Other, *PANSS* Positive and Negative Symptom Scale, *ppm* parts per million, *TOMM* The Test of Memory Malingering, *URICA* University of Rhode Island Change Assessment Scale, *WTAR IQ* Wechsler Test of Adult Reading Intelligence Quotient.

^a^Values are in numbers.

^b^Other, *n* = 1 Indonesian.

^c^Screen session.

### Treatment retention

Kaplan-Meier survival analysis showed no significant differences in retention between groups (log rank test = 1.90, df = 1, *p* = 0.17). Retention was high (~90%, 17/19 completers), with both non-completers assigned to the sham group. For integrity of the blind, participants were no better than chance (~50%) rating rTMS group assignments.

### Cannabis use (primary objective)

Both groups reduced cannabis use from Baseline to Day 28 when considering study completers (active *n* = 9; sham *n* = 8) via self-report and NarcoCheck (Table 2). Approximately 20% of participants abstained from cannabis use, with no group differences.

**Table 2:** Cannabis reduction rates determination outcomes.

Assessment	Active (%)	SE	Lower 95% CI	Upper 95% CI	Sham (%)	SE	Lower 95% CI	Upper 95% CI	Mann–Whitney *U*, *p*-value
GPD	57.97	16.31	26.00	89.95	42.58	17.41	14.14	83.19	*U* = 30, *p* = 0.561
NarcoCheck (ng/mL)	43.78	15.18	14.03	73.53	18.75	23.02	−26.98	69.84	*U* = 33, *p* = 0.473

Reduction rates are comparing Baseline to Day 28 completers resulting in active *n* = 9; sham *n* = 8. A negative reduction rate is indicative of increased cannabis use. The table provides the test statistic, *U* statistic, as well as the asymptotic significance (2-tailed) *p*-value.

*CI* confidence interval, *GPD* grams used per day, *ng/mL* nanograms per milliliter, *SE* standard error.

**Fig. 2:**
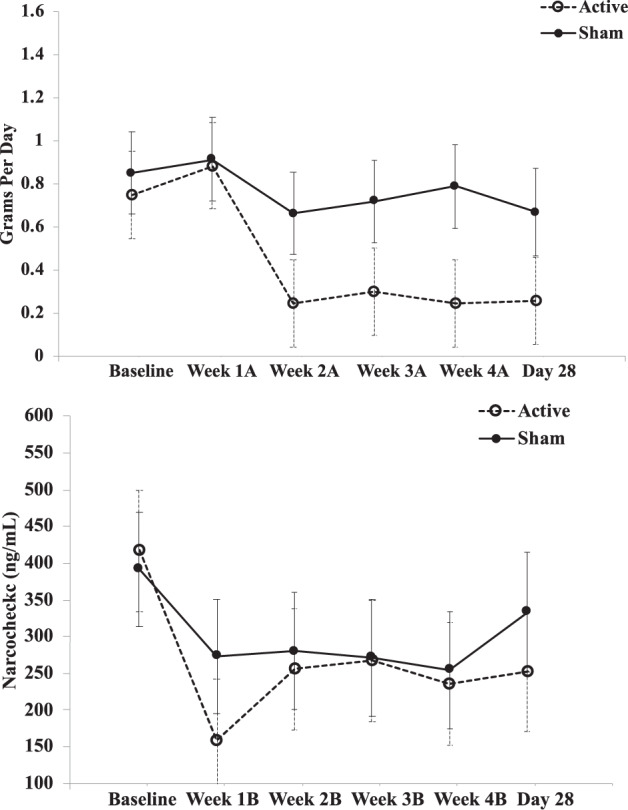
Interaction plots of cannabis use over time between active and sham treatment. Interaction plots of cannabis use with values given as estimated marginal means (EMM) ± standard error are used as error bars. EMM were used in interaction plots as a function to visualize the model at the group level (active and sham, each time point), not at the participant or data point level.

We found no significant difference in change of cannabis use (using GPD, NarcoCheck) from Baseline to Day 28 when comparing active to sham (Table 3). However, contrast estimates were positive, suggesting larger reductions of cannabis use in active versus sham as reflected in both GPD and NarcoCheck (Fig. 2). We found significant main effects of time in GPD and NarcoCheck. Effect sizes between groups were medium for GPD and NarcoCheck (Cohen’s *d* = 0.72 and 0.55, respectively).

**Table 3:** Change in cannabis use from baseline to Day 28 in active compared to sham treatment.

Outcome	Linear Contrast Test	Average trajectory difference: interaction test			Cohen’s *d*
		Treatment * Time	Treatment	Time	
GPD	Estimate = 0.33, df(81.82), *p* = 0.207	F = 1.34, df(81.67), *p* = 0.255	F = 1.98, df(16.55), *p* = 0.177	F = 4.62, df(81.67), *p* = 0.001*	0.72
NarcoCheck (ng/mL)	Estimate = 108.60, df(79.26), *p* = 0.256	F = 0.63, df(79.19), *p* = 0.680	F = 0.14, df(16.97), *p* = 0.711	F = 4.01, df(79.19), *p* = 0.003*	0.55

Positive estimates for the linear contrast indicate more change from Baseline to Day 28 in the Active group. *GPD* grams used per day; *ng/mL* nanograms per milliliter.

### Cannabis craving/withdrawal

No significant difference in MCQ-SV General Factor, Factors 1–2, and 4 scores from Baseline to Day 28 between treatment groups were found (Table 4). Contrast estimates indicated greater reductions in scores in active vs. sham. There were no significant interactions or main effects of treatment, however, significant main effects of time were found (*p* < 0.05).

**Table 4:** Changes in cannabis craving, withdrawal and psychiatric symptoms from baseline to Day 28 in active compared to sham treatment.

Outcome	Linear contrast test	Average trajectory difference: interaction test		
		Treatment * Time	Treatment	Time
MCQ-SV	Estimate = 9.34, df(81.90), *p* = 0.190	F = 0.81, df(81.81), *p* = 0.549	F = 0.25, df(16.74), *p* = 0.624	F = 3.48, df(81.81), *p* = 0.007*
MCQ1	Estimate = 1.92, df(81.53), *p* = 0.334	F = 0.50, df(81.40), *p* = 0.773	F = 0.45, df(16.32), *p* = 0.513	F = 2.59, df(81.40), *p* = 0.031*
MCQ2	Estimate = 1.88, df(81.89), *p* = 0.405	F = 0.84, df(81.77), *p* = 0.525	F = 0.15, df(16.68), *p* = 0.706	F = 3.01, df(81.77), *p* = 0.015*
MCQ3	Estimate = 3.92, df(82), *p* = *0.064*	F = 2.03, df(81.91), *p* = *0.084*	F = 0.003, df(16.84), *p* = 0.960	F = 3.11, df(81.91), *p* = 0.013*
MCQ4	Estimate = 1.52, df(82.09), *p* = 0.484	F = 0.67, df(81.99), *p* = 0.646	F = 0.73, df(16.91), *p* = 0.406	F = 1.97, df(81.99), *p* = *0.091*
MWC	Estimate = −1.10, df(82.18), *p* = 0.586	F = 1.17, df(82.10), *p* = 0.330	F = 2.67, df(17.03), *p* = 0.121	F = 5.23, df(82.10), *p* < 0.0001*
PANSS (Positive)	Estimate = 2.42, df(80.10), *p* = 0.018*	F = 2.05, df(80.08)*, p* = *0.080*	F = 1.39, df(17.02), *p* = 0.255	F = 2.09, df(80.08), *p* = *0.076*
PANSS (Negative)	Estimate = 1.39, df(80.07), *p* = 0.297	F = 0.27, df(80.05), *p* = 0.930	F = 0.19, df(16.96), *p* = 0.665	F = 0.72, df(80.05), *p* = 0.612
PANSS (General)	Estimate = 1.20, df(79.94), *p* = 0.321	F = 2.14, df(79.93), *p* = *0.069*	F = 0.45, df(16.87), *p* = 0.513	F = 0.76, df(79.93), *p* = 0.582
PANSS Total	Estimate = 5.03, df(80.07), *p* = 0.019*	F = 2.79, df(80.06), *p* = 0.023*	F = 1.14, df(17), *p* = 0.301	F = 2.21, df(80.06), *p* = *0.061*
CDSS	Estimate = 0.81, df(82.15), *p* = 0.327	F = 2.15, df(82.08), *p* = *0.068*	F = 0.13, df(17.01), *p* = 0.726	F = 0.65, df(82.08), *p* = 0.651

Outliers flagged by boxplot analysis of residuals were removed and the linear contrast and interaction tests were rerun. Contrast estimates for the linear contrast indicate differences in changes of the outcome from Baseline to Day 28 in the active compared to sham group.

*CDSS* Calgary Depression Scale for Schizophrenia, *MCQ* Marijuana Craving Questionnaire, *MWC* Marijuana Withdrawal Checklist, *PANSS* Positive and Negative Syndrome Scale.

**Fig. 3:**
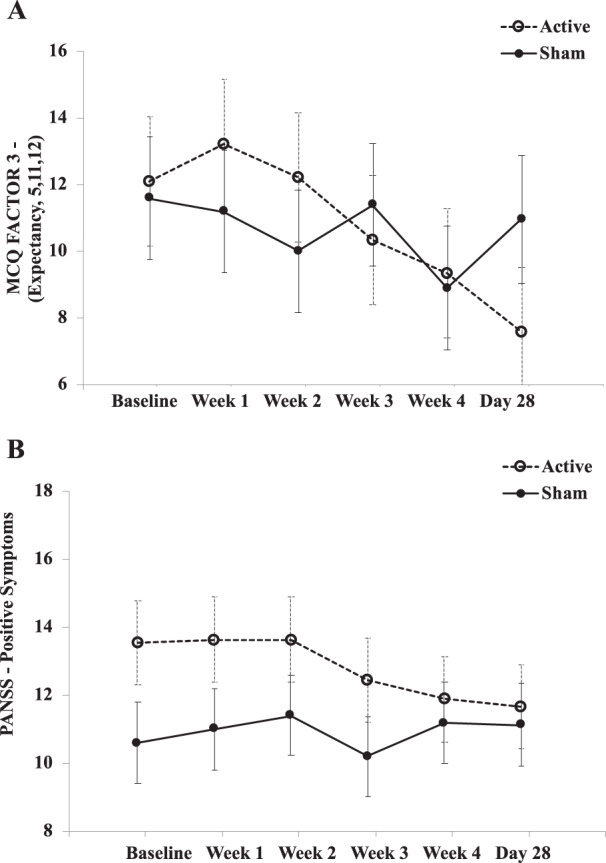
Interaction plots of cannabis craving and psychiatric symptoms over time between active and sham treatment. Interaction plot of cannabis use related symptoms over the course of the trials with values given as estimated marginal means (EMM) ± standard error are used as error bars. EMM were used in interaction plots as a function to visualize the model at the group level (active and sham, each time point), not at the participant or data point level.

There was a trend towards a difference in MCQ Factor 3 score between treatment groups (Estimate = 3.92, df (82), *p* = 0.06) (Fig. 3A); the contrast estimate indicated greater reduction in active vs. sham. The interaction effect trended towards significance (F = 2.03, df (81.91), *p* = 0.08), with a significant main effect of time (F = 3.03, df (86.93), *p* = 0.01), but no main effect of treatment. There were no significant changes from Baseline to Day 28 in the MWC in either the active or sham group.

### Psychiatric symptoms

We found a significant difference in PANSS positive scores from Baseline to Day 28 between active and sham groups (Estimate = 2.42, df (80.10), *p* = 0.02) (Fig. 3B). This contrast estimate indicated greater change in positive symptoms, with a decrease in severity in the active group and an increase in severity in the sham group. However, no significant interaction or main effects were found.

No significant differences emerged for changes in PANSS negative and general scores, and CDSS score from Baseline to Day 28 between treatment groups. A significant difference in PANSS total score was found from Baseline to Day 28 between active and sham (Estimate = 5.03, df (80.07), *p* = 0.02). The contrast estimate indicated a reduction in total score in active vs. sham. A significant interaction effect (F = 2.79, df (80.06), *p* = 0.03) and nearly significant main effect of time (F = 2.21, df (80.06), *p* = 0.06) were found, with no main effect of treatment.

### Cognitive performance

#### Attention

A significant difference was found in hit rate reaction time on the Continuous Performance Test from Baseline to Day 28 between the active and sham groups (Estimate = 6.58, df(15.18), *p* < 0.05) (Table 5). The contrast estimate indicated greater change in sham vs. active, with a greater increase in score in sham, and a smaller decrease in active treatment condition. A significant interaction effect was found (F = 4.64, df(15.18), *p* < 0.05). All other changes/interaction effects in other CPT outcomes were non-significant.

**Table 5:** Change in cognitive performance from baseline to Day 28 in active compared to sham treatment.

	Cognitive assessment variables	Linear contrast test	Average trajectory difference: interaction test		
			Treatment * Time	Treatment	Time
BART	Average Adjusted Pumps	Estimate = −9.32, df(11.87), *p* = 0.152	F = 2.35, df(11.87), *p* = 0.152	F = 0.05, df(15.09), *p* = 0.832	F = 1.42, df(11.87), *p* = 0.257
	Total Money Earned	Estimate = 1.04, df(14.46), *p* = 0.745	F = 0.11, df(14.46), *p* = 0.745	F = 0.75, df(14.90), *p* = 0.399	F = 0.01, df(14.46), *p* = 0.923
HVLT	Total Recall	Estimate = −2, df(16.03), *p* = 0.468	F = 0.55, df(16.03), *p* = 0.468	F = 0.96, df(17.45), *p* = 0.340	F = 0.83, df(16.03), *p* = 0.376
	Delayed Recall	Estimate = −0.21, df(15.91), *p* = 0.866	F = 0.03, df(15.91), *p* = 0.866	F = 0.15, df(17.29), *p* = 0.702	F = 0.03, df(15.91), *p* = 0.866
	% Retention	Estimate = 10.92, df(15.92), *p* = 0.288	F = 1.21, df(15.92), *p* = 0.288	F = 0.04, df(17.30), *p* = 0.838	F = 3.5, df(15.92), *p* = *0.080*
	Discrimination Index	Estimate = −1.37, df(16.76), *p* = 0.248	F = 1.43, df(16.76), *p* = 0.248	F = 0.98, df(17.56), *p* = 0.335	F = 0.001, df(16.76), *p* = 0.974
SDR	30 s Delay	Estimate = 4.70, df(15.87), *p* = 0.611	F = 0.27, df(15.87), *p* = 0.611	F = 0.57, df(17.66), *p* = 0.461	F = 0.10, df(15.87), *p* = 0.755
TMT	Trial A	Estimate = 1.01, df(15.22), *p* = 0.665	F = 0.20, df(15.22), *p* = 0.665	F = 0.37, df(17.05), *p* = 0.549	F = 15.22, df(16.96), *p* = 0.001*
	Trial B	Estimate = −5.11, df(15.56), *p* = 0.716	F = 0.14, df(15.56), *p* = 0.716	F = 0.36, df(17.25), *p* = 0.554	F = 0.11, df(15.56), *p* = 0.748
Digit Span	Forward	Estimate = 0.25, df(15.78), *p* = 0.844	F = 0.04, df(15.78), *p* = 0.844	F = 0.07, df(16.98), *p* = 0.799	F = 1.22, df(15.78), *p* = 0.285
	Backward	Estimate = −0.23, df(15.24), *p* = 0.807	F = 0.06, df(15.24), *p* = 0.807	F = 0.38, df(16.77), *p* = 0.544	F = 1.40, df(15.24), *p* = 0.255
	Total	Estimate = −0.37, df(15.64), *p* = 0.806	F = 0.06, df(15.64), *p* = 0.806	F = 0.33, df(17.27), *p* = 0.571	F = 4.04, df(15.64), *p* = 0.062
CpT	% Correct Hits	Estimate = −1.05, df(12.41), *p* = 0.242	F = 1.51, df(12.41), *p* = 0.242	F = 0.001, df(14.99), *p* = 0.974	F = 0.37, df(12.41), *p* = 0.557
	% Omissions	Estimate = 7.37, df(12.04), *p* = 0.385	F = 0.81, df(12.04), *p* = 0.385	F = 0.07, df(14.59), *p* = 0.790	F = 0.30, df(12.04), *p* = 0.591
	% Commissions	Estimate = −3.20, df(14.14), *p* = 0.469	F = 0.55, df(14.14), *p* = 0.469	*F* = 4.49, df(15.89), *p* = *0.050*	F = 0.86, df(14.14), *p* = 0.371
	Hit Reaction Time	Estimate = 6.58, df(15.18), *p* = 0.048*	F = 4.64, df(15.18), *p* = 0.048*	F = 2.19, df(17.58), *p* = 0.157	F = 0.58, df(15.18), *p* = 0.457
	Variability	Estimate = −14.91, df(14.70), *p* = 0.044*	F = 4.85, df(14.70), *p* = 0.044*	F = 0.19, df(16.90), *p* = 0.672	F = 0.07 df(14.70), *p* = 0.796
	Attentiveness	Estimate = −2.65, df(14.49), *p* = 0.493	F = 0.49, df(14.49), *p* = 0.493	F = 6.01, df(16.75), *p* = 0.025*	F = 0.11, df(14.49), *p* = 0.746
Grooved pegboard	Dominant Hand	Estimate = −12.53, df(15.68), *p* = 0.152	F = 2.27, df(15.68), *p* = 0.152	F = 0.03, df(17.24), *p* = 0.874	F = 0.06, df(15.68), *p* = 0.812
	Dominant Hand # Dropped	Estimate = 0.09, df(16.94), *p* = 0.855	F = 0.04, df(16.94), *p* = 0.855	F = 0.70, df(17.18), *p* = 0.415	F = 0.04, df(16.94), *p* = 0.855
	Non-Dominant Hand	Estimate = −24.26, df(15.71), *p* = 0.044*	F = 4.79, df(15.71), *p* = 0.044*	F = 0.18, df(17.24), *p* = 0.681	F = 0.06, df(15.71), *p* = 0.805
	Non-Dominant Hand # Dropped	Estimate = −0.56, df(16.54), *p* = 0.337	F = 0.98, df(16.54), *p* = 0.337	F = 1.39, df(17.24), *p* = 0.254	F = 1.81, df(16.54), *p* = 0.196
KDDT	Ln(k) Small Reward	Estimate = −1.37, df(10.1), *p* = 0.273	F = 1.35, df(10.1), *p* = 0.273	F = 0.53, df(10.7), *p* = 0.484	F = 3.37, df(10.1), *p* = 0.096
	Ln(k) Medium Reward	Estimate = −2.05 df(10), *p* = 0.152	F = 2.4, df(10), *p* = 0.152	F = 0.31, df(10), *p* = 0.589	F = 1.83, df(10), *p* = 0.206
	Ln(k) Large Reward	Estimate = −3.44 df(24), *p* = *0.059*	F = 3.92, df(24), *p* = *0.059*	F = 0.76, df(24), *p* = 0.391	F = 5.23, df(24), *p* = 0.031*
	Ln Geometric k	Estimate = −1.54, df(11.5), *p* = 0.175	F = 2.09, df(11.5), *p* = 0.175	F = 0.57, df(11.74), *p* = 0.464	F = 3.17, df(11.5), *p* = 0.101
TOL	Total Moves	Estimate = −2.5, df(6), *p* = 0.784	F = 0.057, df(6), *p* = 0.819	F = 0.93, df(6), *p* = 0.372	F = 10.44, df(6), *p* = 0.018*
	Total Correct	Estimate = 3.5, df(6), *p* = 0.801	F = 0.07, df(6), *p* = 0.801	F = 0.11, df(6), *p* = 0.756	F = 1.73, df(6), *p* = 0.237
	Total Time Violation	Estimate = 5, df(12), *p* = 0.722	F = 0.13, df(12), *p* = 0.722	F = 1.36, df(12), *p* = 0.267	F = 1.36, df(12), *p* = 0.267
	Total Initiation Time	Estimate = −7.0, df(6), *p* = 0.241	F = 1.69, df(6), *p* = 0.241	F = 0.01, df(6), *p* = 0.923	F = 0.86, df(6), *p* = 0.389
	Total Execution Time	Estimate = 4, df(6), *p* = 0.398	F = 0.83, df(6), *p* = 0.398	F = 0.86, df(6), *p* = 0.389	F = 10.14, df(6), *p* = 0.019*
	Total Problem-Solving Time	Estimate = 8, df(6), *p* = 0.218	F = 1.89, df(6), *p* = 0.218	F = 0.43, df(6), *p* = 0.54	F = 1.44, df(6), *p* = 0.274
MMN	Amplitude	Estimate = −0.32, df(12.33), *p* = 0.557	F = 0.37, df(12.33), *p* = 0.557	F = 1.32, df(14.57), *p* = 0.268	F = 2.46, df(12.33), *p* = 0.142

Outliers flagged by boxplot analysis of residuals were removed and the linear contrast and interaction tests were rerun. Contrast estimates for the linear contrast indicate differences in changes of the outcome from Baseline to Day 28 in the active compared to sham group.

*BART* Balloon Analogue Risk Task, *CPT* Continuous Performance Test, *HVLT* Hopkins Verbal Learning Test-Revised, *KDDT* Kirby Delay Discounting Task, *MMN* Mismatch negativity amplitude, *SDR* Spatial Delayed Response, *SS* standardized score, *TMT* Trail Making Test, TOL Tower of London.

#### Motor function

There was a significant difference in changes of non-dominant hand total time (Grooved Pegboard^47^) from Baseline to Day 28 between treatment groups (Estimate = −24.26, df(15.71), *p* < 0.05). The contrast estimate demonstrated greater and opposite direction of change over time in active vs. sham, with an increase in time in active and decrease in sham. A significant interaction effect was found (F = 4.79, df(15.71), *p* < 0.05).

Across other cognitive tests, we found no significant differences in changes from Baseline to Day 28, and no significant interactions.

### Tobacco use

**Fig. 4:**
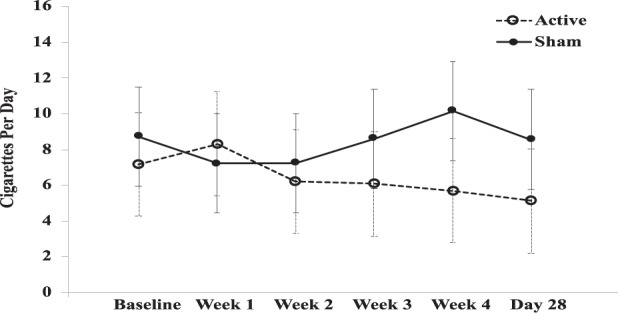
Interaction plot of cigarette use over time between active and sham treatment. Interaction plot of cigarette use over the course of the trial with values given as estimated marginal means (EMM) ± standard error was used as error bars. EMM were used in interaction plots as a function to visualize the model at the group level (active and sham, each time point), not at the participant or data point level.

Contrast estimates indicated greater reduction in self-reported cigarettes/day in active vs. sham (Fig. 4), with a significant time x treatment interaction (F = 3.15, df(82.02), *p* = 0.01).

### Safety profile

No participant reported more than two AEs during the trial (Table 6). Total AEs reported were grouped: zero AEs reported, 1 AE reported, and 2 AEs reported. No association between total AEs and treatment was found (*χ*^2^) = 1.55, *p* = 0.46).

**Table 6:** Adverse events reported by rTMS technicians during treatment weeks.

Treatment	Treatment Week	Treatment #	AE	Severity	Attribution
Active	1	2	Headache	Mild	Possibly related to study device
		3		Mild	Possibly related
	2	9	Neck Pain	Moderate	Possibly related
	3	11	Headache	Mild	Possibly related
		12		Mild	Possibly related
		15		Moderate	Possibly related to study device
	4	16	Headache	Moderate	Possibly related
		19	Dizziness	Moderate	Probably not related
Sham	1	2	Headache	Mild	Possibly related
	2	9	Sinus Infection	Mild	Probably not related
	3	12	Tinnitus	Mild	Possibly related to study device
		12	Headache	Mild	Possibly related
		13	Application Site Pain (electrode location)	Moderate	Most probably related

Individual and all adverse events reported by rTMS technicians by treatment group, week, and session number during the entire duration of the treatment trial.

*AE* adverse event.

## Discussion

This is the first randomized, double-blind, parallel-group, sham-controlled study on the efficacy and safety of high-frequency rTMS for cannabis use in schizophrenia. Our goal was to determine the effects of 4-weeks of active (20-Hz) versus sham rTMS treatment directed bilaterally to the DLPFC on cannabis use outcomes. Based on two different measures (e.g., self-report, semi-quantitative urine toxicology), greater reductions in cannabis use were found in the active versus the sham group; however, these results were not significant. However, the magnitude of standardized change (Cohen’s *d*-like) between treatment groups indicated medium effects for decreases in self-reported cannabis use (*d* = 0.72) and Narcochek semi-quantitative urine toxicology (*d* = 0.55) in the active group compared to the sham group, which are notably in the clinically significant range. Thus, our preliminary findings are promising and in the predicted direction.

A case series (*N* = 3) suggested that rTMS directed to bilateral DLPFC for 20 sessions may reduced cannabis use and craving, with a large effect size (Cohen’s *d* = 1.2)^42^. Since these effects of rTMS may be mediated by targeting specific brain circuits^35^, it was critical to evaluate cannabis craving/withdrawal symptoms. Contrast estimates demonstrated greater reduction in craving symptoms (MCQ: Total, Factor 1–4) in the active group compared to the sham group. We also found a trend towards a group difference in change in MCQ Factor 3 (e.g., appetitive states) from Baseline to Day 28. Thus, active rTMS may lower anticipation of positive cannabis outcomes^48^. It is unclear why this specific aspect of craving was reduced by rTMS. Given that anticipation of drugs involves release of ventral tegmental dopamine resulting in strong motivation to seek substances^49^, rTMS to the DLPFC may facilitate dopamine and glutamate release in subcortical regions (e.g., nucleus accumbens), thereby reducing anticipation of cannabis use^39^. This finding is consistent with our previous study using the same paradigm in tobacco users resulting in greater reduction of anticipation of positive effects of tobacco, and relief from withdrawal in active versus sham groups^44^. Thus, reduced appetitive effects for cannabis implicates promising therapeutic effects of rTMS on reward circuitry.

Similar non-significant reductions in craving have been found by another multiple-session active rTMS trial examining effects of treatment for cannabis craving^42^. In a cross-over design evaluating effects of a single active rTMS session versus sham on cravings (MCQ) in CUD, Sahlem et al. found no significant craving reductions^41^. Notably, when comparing our pre- and post-ratings in the active group to this study’s results, our findings demonstrated substantial reductions across all factors of the MCQ (~35% versus ~2%, respectively). This suggests that multiple rTMS sessions are required to achieve therapeutic effects.

We found that active rTMS significantly improved positive and total symptoms from Baseline to Day 28. Whether this effect was due to rTMS versus other factors, such as reduced cannabis use or improved cognition, is less clear. However, in our previous study investigating the effects of cannabis abstinence on PANSS symptoms in schizophrenia patients, we found that there was no change in positive or total symptoms associated with cannabis abstinence or cognitive improvement^50^, suggesting that in this study rTMS is likely driving the effect of improved symptomatology in these patients. In support of this, changes in cannabis use did not predict changes in positive and total symptoms within each treatment group. Given positive symptoms are associated with hyperactivity of the left temporoparietal cortex (TPC), low frequency rTMS may improve^51^, while high-frequency rTMS may worsen positive symptoms^52^. Moreover, two meta-analyses found no improvement in positive symptoms with low frequency rTMS targeting the left TPC^53,54^, but found high-frequency rTMS targeting the left DLPFC improved positive symptoms^55^. Thus, given the negative findings of previous studies using low-frequency to the left TPC, our results suggest that to treat positive symptoms it may be effective to target other brain regions (e.g., DLPFC) with high-frequency rTMS. One explanation is that high-frequency rTMS activates fronto-temporal circuits via transsynaptic activation^56^. Another consideration is the impact of high-frequency rTMS on glutamatergic pathways from the PFC to the striatum, activating hypofunctional NMDA glutamatergic neurotransmission, leading to reduced glutamatergic neuron firing, and attenuating dopamine release^57^. rTMS may also improve the pathophysiology of CUD by upregulating CB_1_ receptors. rTMS elevates CB_1_ activity in rodent models^58^, which may result in improved feedback inhibition and downstream regulation of hyperactive midbrain dopamine neurons^59,60^.

The majority of the cognitive outcomes (e.g., verbal learning and memory, working memory, impulsivity, executive function) were not altered by rTMS. The reductions in cannabis use we observed may have led to acute withdrawal that was not attenuated by active rTMS treatment, and may have masked improvement in cognition. This finding has been previously found in comorbid schizophrenia and tobacco use disorder^45^. Future studies with larger samples should conduct further analyses and control for such potential confounding relationships (e.g., active rTMS being linked to reductions in cannabis use and improvements in cognition via indirect neurobiological improvements). We found that active rTMS significantly improved sustained attention, as indicated by a faster hit reaction time on CPT, consistent with previous work^61,62^. Notably, there was no increase in commission errors indicating that the prior effect was not due to greater impulsivity, but rather improved sustained attention in the active group^61,62^.

We examined the effects of rTMS on tobacco use, given that co-use of cannabis and tobacco is high in schizophrenia^63^. We expected a compensatory increase in tobacco use with reduced cannabis use, based on our previous studies^64,65^. We found a significant difference in the trajectory of tobacco use between active and sham groups. There was a reduction of tobacco use in the active group during treatment (~2 CPD), but a transient increase in the sham group. These results support our previous findings where 28-days of cannabis abstinence in schizophrenia resulted in a transient increase in tobacco consumption^65^. Thus, active rTMS treatment may attenuate this compensatory tobacco increase, suggesting that rTMS has broader anti-drug effects.

Survival analysis demonstrated no differences in retention rates between treatments. Presence of minor common side effects (e.g., headache) reported in both treatments did not result in discontinuation by any participant. There are only two other high-frequency rTMS trials among CUD. The first trial used one rTMS session and was found safe^41^. The second trial conducted by this group was a case series with 20 sessions of high-frequency rTMS administered over two weeks^42^. Thus, high-frequency rTMS appears to be well-tolerated by cannabis users with and without schizophrenia.

### Limitations and strengths

Our study sample size was underpowered and thus our results should be considered preliminary, requiring replication in larger samples with more female participants for enhanced generalizability of findings. Use of a multisite design may aid in recruitment of a larger sample. Other limitations included the use of a semi-quantitative urine drug assay as an objective index of cannabis reduction/abstinence. While Narcocheck has a more sensitive threshold for detecting THC-COOH than most qualitative drug tests (<18 ng/mL versus 50 ng/mL), even with repeated testing results cannot predict whether abstinence was sustained throughout the 4-week trial. Future studies should obtain twice weekly creatinine-corrected quantitative THC-COOH levels using chromatographic and mass spectrometric methods. Applying a mathematical model (e.g., Schwilke et al.^66^) to these THC-COOH levels can determine, with a high degree of certainty, if cannabis abstinence was indeed sustained^67^.

However, there were several strengths. We used a prospective, sham-controlled study design and were able to monitor differences in the effects of rTMS on cannabis use both within and between participants using self-reported use and semi-quantitative urine toxicology. The integrity of the blind was preserved, eliminating bias in treatment assignment. The nature of our rTMS paradigm is a strength given that use of sham stimulation mimics the sensations of active rTMS. We used a validated rTMS paradigm^44^ with rigorous screening and well-matched treatment groups. Finally, contingent payments reinforced rTMS session attendance resulting in high-retention (~90%), maximizing rTMS effects. These preliminary findings provide important insights into the therapeutic potential of rTMS to the DLPFC in people with co-occurring schizophrenia and CUD.

## Methods

### Participants

Inclusion criteria included: (1) male/female outpatients, 18–55 years; (2) DSM-5 diagnoses of schizophrenia/schizoaffective disorder and CUD using the Structured Clinical Interview for DSM-5 (SCID-5); 3) stable antipsychotic dose (>1 month); (4) scoring <70 on the Positive and Negative Syndrome Scale for Schizophrenia (PANSS);^68^ (5) scoring ≥12 on the Cannabis Use Disorder Identification Test–Revised (CUDIT-R);^69^ (6) treatment-seeking for CUD, based on a score ≥7 on the Marijuana Contemplation Ladder;^70^ (7) Full-Scale IQ score ≥80 on Wechsler Test of Adult Reading (WTAR);^71^ (8) positive urine for THC (MEDTOX^TM^).

Exclusions were: (1) urine positive for drugs besides THC; (2) DSM-5 diagnoses for alcohol, substance/polysubstance use disorder (other than cannabis, caffeine, tobacco) in the past six months; (3) active suicidal ideation or self-harm (on Columbia-Suicide Severity Rating Scale (C-SSRS)^72^); (4) previous head injury with loss of consciousness (>5 min) and hospitalization; (5) major neurological or medical illness including seizure disorder or syncope; (6) metallic implants; (7) previous rTMS.

### Experimental procedures

The study protocol was approved by the CAMH Research Ethics Board (REB; #017/2017). The study was conducted at CAMH, a tertiary mental health hospital and conducted from October, 2017 through March, 2020. The study was registered at ClinicalTrials.gov (NCT03189810).

**Fig. 5:**
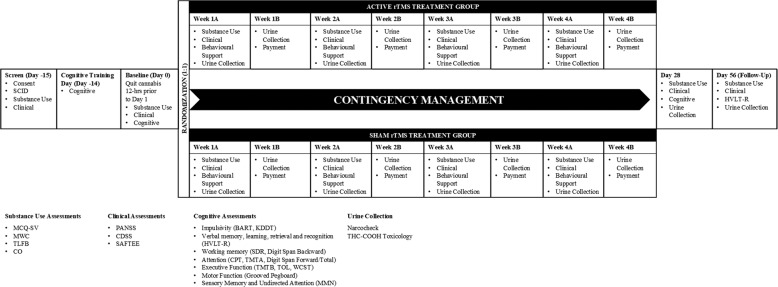
Study timeline and assessment schedule. Participants found eligible after an in-person screen, completed Cognitive Training Day (exclusive of MMN), followed by a 2-week lead-in phase including a Baseline (Day 0) in which a cognitive battery, MMN, and clinical and substance use outcomes were assessed. They were then randomized into either active or sham rTMS treatment, Monday to Friday over 4 consecutive weeks, during which assessments were completed to monitor clinical symptoms and substance use. Behavioral support was also provided at these sessions. Contingency management was used to reinforce participants’ attendance. The same battery of assessments as on Baseline was then completed on Day 28 (post session). A brief session was also completed on Day 56 (Follow-Up session). CDSS, Calgary Depression Scale for Schizophrenia; CPT, Continuous Performance Test; F/U, Follow-Up; HVLT, Hopkins Verbal Learning Test-Revised; KDDT, Kirby Delay Discounting Task; MMN, Mismatch negativity; PANSS, Positive and Negative Symptom Scale; rTMS, repetitive transcranial magnetic stimulation; SCID, Structured Clinical Interview; SDR, Spatial Delayed Response Test; TMT, Trail Making Test; WCST, Wisconsin Card Sorting Test.

Participants were screened using a standardized phone interview. Eligible participants were invited for an in-person screen, completing study consent, a comprehension quiz on study procedures (score ≥80% required) and diagnostic interview. Eligible participants were invited to a cognitive training session to become familiarized with cognitive tasks. After a 2-week lead-in, participants completed a baseline session consisting of a cognitive battery, substance-related and psychiatric symptom assessments. A 12-h period of cannabis abstinence prior to baseline testing was implemented to ensure participants were not intoxicated on cannabis prior to baseline cognitive and clinical assessments. This was verified by clinical assessment, as well as measures of craving (MCQ) and withdrawal (MWC). Participants were then randomized (1:1) to receive either active or sham rTMS administered Monday–Friday (M–F) over 28 days (5x/week, M–F) (Fig. 5). To maximize active versus sham rTMS effects, CM consisting of progressive payments at the end of each rTMS treatment for reinforcing session attendance were made; missed appointments received no compensation. The payment schedule was as follows: $50 on Week 1B, $65 on Week 2B, $85 on Week 3B and $100 on Week 4 (Day 28). Thus, attendance at all rTMS sessions earned participants a total of $300.

Behavioral support sessions and psychiatric and substance use assessments were completed weekly on Mondays (Weeks 1–4). Urine samples were collected twice weekly (Mondays and Thursdays). The cognitive battery, clinical and substance-related assessments were then administered at Days 28, and Day 56 follow-up for cognitive, clinical and substance use outcomes.

### Substance use measures

Severity of tobacco smoking was assessed using the Fagerstrom Test for Nicotine Dependence (FTND)^73^ and verified by an expired breath carbon monoxide (Smokerlyzer®) test. Joint-Years^74^ were calculated to assess cannabis lifetime exposure. One Joint-Year equated to smoking one joint per day for one year. The Alcohol Use Disorders Identification Test^75^ assessed problematic alcohol use. Marijuana Withdrawal Craving (MWC) assessment^76^ monitored cannabis withdrawal symptoms, the Marijuana Craving Questionnaire (MCQ-short version)^77^ assessed cannabis craving, and the TLFB^78^ assessed self-reported substance use (cannabis, alcohol, tobacco and caffeine) from the previous 7-days; all were administered at Baseline, Week 1–4, and Day 56.

### Monitoring of cannabis use

Cannabis reduction/abstinence was monitored weekly by two methods: 1) self-reported use each Monday using the TLFB-cannabis (grams per day, GPD); and 2) NarcoCheck® (Villejuif, France) semi-quantitative THC Pre-Dosage Test administered on Mondays and Thursdays during the trial. Narcochek is an Enzyme Multiplied Immunoassay Technique (EMIT) which was used to determine point of care semi-quantitative THC-COOH (a metabolite of THC) levels twice weekly during the 4-week rTMS trial to monitor on-going cannabis use reductions and abstinence. The semi-quantitative urinalysis of THC-COOH has five levels of THC-COOH: < 18 ng/ml (0), 50 ng/ml (1+), 150 ng/ml (2+), 300 ng/ml (3+) and 600 ng/ml (4+). The <18 ng/ml level is considered to be consistent with cannabis abstinence^79^.

### Clinical measures

The PANSS^68^ measured psychotic symptom severity. The Calgary Depression Scale for Schizophrenia (CDSS)^80^ assessed depression independent of negative and extrapyramidal symptoms. Both were administered at Baseline, Week 1–4, and Day 56.

### Behavioral support

A combination of psychoeducation, motivational interviewing, coping skills, and withdrawal management was administered weekly (<15 min duration) to assist participants achieve cannabis reduction/abstinence^81^.

### Cognitive measures

Cognitive assessments were completed on the Cognitive Training Day, Baseline, and Day 28. The order of assessments were counterbalanced across participants and included paper/pencil and computerized tasks described previously^19^. Additionally, the Tower of London (TOL)^82^ assessing executive functioning, and mismatch negativity (MMN^83^) a component of the event-related potential assessing pre-attentive processing, specifically auditory change detection and reflects the detection of novelty^84^, were administered at Baseline and Day 28.

### rTMS treatment

Participants were randomly assigned to active or sham rTMS for 20 sessions, 5 days per week over four weeks (M–F). Missing two consecutive treatments within the same week resulted in withdrawal from the trial. Investigators and participants were blind to treatment assignment. The integrity of the blind was assessed by asking participants at trial endpoint which treatment allocation they believed was administered.

rTMS was administered using a MagProX100 (MagVenture, Farum, Denmark) equipped with a B65 active/placebo coil. rTMS stimulation sites (left and right DLPFC), were localized using electroencephalography with F5/F6 electrodes^85^. The order of bilateral stimulation was counterbalanced. Resting motor threshold (RMT) for each participant was determined during the first treatment session^86^. rTMS was delivered at an intensity of 90% of the RMT, frequency of 20-Hz (25 trains, 30 pulses per train, 30 s intertrain intervals). rTMS technicians monitored any adverse events (AEs).

### Statistical analysis

All data were entered into REDCap and analyzed using Statistical Program for Social Sciences (SPSS, v.26). The analysis was conducted on an intention to treat (ITT) basis. All tests were two-tailed and conducted with *p* < 0.05. Trend level significance was set at *p* = 0.05–0.09. No adjustments for multiple comparisons were made for the exploratory study. Demographic, clinical, and substance outcomes were calculated using mean ± SE with independent t-tests; categorical data was analyzed using Fischer’s Exact or Chi-Square tests. Kaplan-Meier survival analysis examined differences in retention between groups^87^.

Cannabis reductions were calculated by determining differences between Baseline and Day 28 within each treatment group (GPD); NarcoCheck). The non-parametric Mann–Whitney *U*-Test compared reduction rates between groups, since the percentage change was not normally distributed.

Primary analyses used linear mixed-effects models (LMM) testing change from Baseline to Day 28 between treatment groups; missing data was managed using maximum likelihood estimation. The linear contrast estimate was the difference in change from Baseline to Day 28 (i.e., active minus sham change). Positive and negative contrast estimates indicated direction and scale of change in the active vs. sham group. Outliers in boxplot analysis of residuals (>3 SD from mean) were removed and linear contrast and interaction tests were rerun.

Secondary analysis tested average trajectories of primary and secondary objectives, using Treatment × Time interactions. If time or treatment alone were significant, the linear contrast test was rerun to confirm the effect.

We calculated Cohen’s *d*-like standardized change by dividing estimated change in treatment groups (derived from mixed effect models using estimated marginal means) by pooled standard deviation of change across both treatments, estimated after calculating individual level change.

### Reporting summary

Further information on research design is available in the Nature Research Reporting Summary linked to this article.

## Supplementary information

REPORTING SUMMARY

## Data Availability

The data supporting the findings of this study are not publicly available due to ethical restrictions for protecting participants’ confidentiality and privacy but are accessible from the corresponding author on reasonable request with the approval of the Institutional Review Board of CAMH.

## Code availability

No custom code or mathematical algorithm was used for this study.
